# Armodafinil versus Modafinil in Patients of Excessive Sleepiness Associated with Shift Work Sleep Disorder: A Randomized Double Blind Multicentric Clinical Trial

**DOI:** 10.1155/2011/514351

**Published:** 2011-06-01

**Authors:** D. V. Tembe, A. Dhavale, H. Desai, D. N. Mane, S. K. Raut, G. Dhingra, U. Sardesai, S. Saoji, M. Rohra, V. G. Shinde, M. Padsalge, A. Paliwal, K. Abbasi, P. Devnani, S. Papinwar, S. Phadke, H. Mehta, V. Bhailume

**Affiliations:** ^1^Manovedh Clinic, Karad 415110, India; ^2^A.J. Medical Care Pvt. Ltd., Mulshi, Pune 411027, India; ^3^Dr. Hemang Desai's Clinic, Ahmedabad 380007, India; ^4^Noble Hospital, Pune 411013, India; ^5^Dhanvantari Hospital, Pune 411028, India; ^6^Dr. Gurpreet Dhingra's Clinic, Santacruz (W), Mumbai 400049, India; ^7^Samvedana, Indore 452003, India; ^8^Saoji-Tupkari Hospital, Aurangabad 431005, India; ^9^Dr. Manoj Rohra's Clinic, Santacruz (W), Mumbai 400049, India; ^10^Shinde's Medicare Hospital Pvt. Ltd., Andheri, Mumbai 400053, India; ^11^Shivam Clinic and Research Centre, Nerul, Navi Mumbai 400706, India; ^12^Dr. Abhay Paliwal's Clinic, Indore 452001, India; ^13^Jeswani Hospital, Nagpur 440002, India; ^14^Sleep Disorder Clinic, Khar, Mumbai 40052, India; ^15^Papinwar Hospital, Aurangabad, 431001, India; ^16^Dr. Sanjay Phadke's Clinic, Kothrud, Pune 411038, India; ^17^Dr. Hemal Mehta's Clinic, Vile Parle (E), Mumbai 400057, India; ^18^Dr. Vilas Bhailume's Clinic, Karve Nagar, Pune 411052, India

## Abstract

*Aim*. To compare the efficacy and safety of armodafinil, the R-enantiomer of modafinil, with modafinil in patients of shift work sleep disorder (SWSD). *Material and Methods*. This was a 12-week, randomized, comparative, double-blind, multicentric, parallel-group study in 211 patients of SWSD, receiving armodafinil (150 mg) or modafinil (200 mg) one hour prior to the night shift. *Outcome Measures*. Efficacy was assessed by change in stanford sleepiness score (SSS) by at least 2 grades (responder) and global assessment for efficacy. Safety was assessed by incidence of adverse events, change in laboratory parameters, ECG, and global assessment of tolerability. *Results*. Both modafinil and armodafinil significantly improved sleepiness mean grades as compared to baseline (*P* < .0001). Responder rates with armodafinil (72.12%) and modafinil (74.29%) were comparable (*P* = .76). Adverse event incidences were comparable. *Conclusion*. Armodafinil was found to be safe and effective in the treatment of SWSD in Indian patients. The study did not demonstrate any difference in efficacy and safety of armodafinil 150 mg and modafinil 200 mg.

## 1. Introduction 

A significant proportion of employed individuals in India work during night hours. This causes misalignment between the sleep and wake propensities that are controlled by hypothalamic circadian pacemaker [1] and results into shift work sleep disorder (SWSD). The reported incidence of SWSD in India is about 44.8% of night-shift workers and 35.8% of rotating workers [2]. SWSD is characterized by persistent excessive sleepiness during night work and insomnia when attempting sleep in the daytime [3]. Individuals with SWSD have significantly higher incidence of sleepiness-related accidents, absenteeism, depression, and missed family and social activities as compared with other night-shift workers [2]. It is also associated with higher incidence of ulcers, cardiovascular disease, and deficit in cognition and psychomotor performance [4, 5]. 

The pharmacological management of SWSD involves treatment with modafinil that has been shown to improve wakefulness and ability to sustain attention in these patients. However, despite the half-life of 15 hours, the wakefulness promoting effect of modafinil is found to be ill-sustained in the last one third of night shift hours [6]. The lack of efficacy in the early morning hours and undue patient confidence in the drug can result into excessive sleepiness while commuting home. This can increase the chances of sleepiness-related accidents. Armodafinil, the chirally pure R-enantiomer of modafinil, approved by US FDA in 2007 has half-life (*t*_1/2_ = 15 hours) three times longer than its S-enantiomer (*t*_1/2_ = 3 hours) [7]. Despite the same half lives, comparison of the equivalent (200 mg) doses of modafinil and armodafinil, in humans has revealed that armodafinil sustains higher plasma concentrations 6–14 hrs postadministration than that of racemic modafinil with longer maintenance of wakefulness [8–10]. 

This was a randomized, comparative, double-blind, and multicentric study comparing the effects of modafinil 200 mg with armodafinil 150 mg in Indian patients of SWSD. To our knowledge, this is the first comparative study in SWSD patients.

## 2. Materials and Methods

### 2.1. Study Design and Approvals

Prior approval was obtained from Drug Controller General of India (DCGI) and appropriate ethics committees. The study was conducted in accordance of Good Clinical Practice guidelines (issued by Central Drugs Standard Control Organization, Government of India) and according to the Declaration of Helsinki. The trial was registered at the Clinical Trials Registry, India (http://www.ctri.in/).

### 2.2. Patients

After obtaining written informed consent, patients of either sex, aged between 18 and 60 years, attending outpatient clinics of the authors, and suffering from excessive sleepiness associated with SWSD (assessed basis patient' primary complaint and using the diagnostic criteria adopted from international classification for sleep disorders [3] (Table 1)) were enrolled. Patients were working at least five night shifts every month for 12 hours or less, with 6 hours or more working between 10 p.m. and 8 a.m. and at least three shifts occurring consecutively. The major exclusion criteria were patients with significant liver or kidney or heart diseases, patients with clinically significant, uncontrolled psychiatric or medical condition, patients with known history of hypersensitivity to formulation, patients operating an automobile or hazardous machinery, caffeine consumption averaging more than 600 mg/day within 1 week of baseline, use of other concomitant medications which inhibit, induce, or are metabolized by CYP450, patients using sedative or CNS acting drugs or medication liable to affect outcome of the study (e.g., antihistamines, selective serotonin reuptake inhibitors, tricyclic antidepressants, lithium, anti-psychotics, anticonvulsants, monoamine oxidase inhibitor, benzodiazepines, psychostimulants, and anticoagulants), pregnant and lactating mothers, females of reproductive age and expecting pregnancy or using steroidal contraception, and patients with alcohol or drug abuse.

### 2.3. Study Design and Medications

This was a multi-centric, randomized, comparative, and double-blind parallel group, clinical trial conducted over 18 sites across India. Randomization in blocks of ten was carried out in 1 : 1 ratio for test and reference products online at http://www.randomization.com/. Patients received orally either armodafinil 150 mg tablet (Emcure Pharmaceuticals Ltd., India) or modafinil 200 mg tablet (from commercial source) one hour prior to start of every night shift for 12 weeks. Coprescriptions not interfering with the study drug evaluation were allowed. The test formulation was earlier found to be bioequivalent to the US FDA-approved formulation of armodafinil, in 26 healthy Indian volunteers [11]. The tablets of armodafinil and modafinil were identical in shape, size, and color and were dispensed in coded, identical, and opaque packs to conceal identity and maintain blinding.

### 2.4. Efficacy Assessment

Patients were evaluated for sleepiness score based on Stanford sleepiness Scale (SSS) at baseline, 4 weeks, 8 weeks, and 12 weeks [12]. All the assessments were done in the morning hours at the end of three consecutive night shifts. The primary efficacy endpoint was proportion of patients showing at least 2 grades of improvement (responder) based on SSS in both groups. The other efficacy variables included improvement in mean SSS grades compared to baseline, compliance to therapy, and patients' as well as physicians' global assessment for efficacy. Global assessment of efficacy was performed using the following grades: (i) excellent = reduction of >75% of symptoms, (ii) good = reduction of 51–75% of symptoms, (iii) fair = reduction of 26–50% of symptoms, and (iv) poor = no improvement or reduction in <25% of symptoms. Patients' compliance to the therapy was calculated in percentage by using following formula: (number of tablets actually taken ×100)/number of tablets supposed to be taken.

### 2.5. Safety Assessment

A general and detailed systemic examination was performed for all patients during each study visit. Blood samples were collected at baseline and at the end of the study for complete hemograms, liver function tests, renal function tests, lipid profile, and fasting blood glucose levels. Electrocardiograms were performed for all patients at baseline and at the end of the study. Tolerability was assessed by recording patients' global assessment about the tolerability of the drug and percent of the patients experiencing any drug-related adverse events. The global assessment of tolerability was performed using following grades: (i) excellent = no adverse drug reaction, (ii) good = mild adverse drug reaction but no interference with normal lifestyle, (iii) fair = mild adverse drug reaction which interference with normal lifestyle. However, benefits of drug therapy outweigh the inconvenience, (iv) poor = drug withdrawn.

### 2.6. Statistical Analysis

Prestudy calculations showed that a sample size of 100 in each group would have 80% power to detect a difference of at least 19% in responder rate with a significance level (alpha) of.05 (two tailed). Demographic and baseline characteristics were summarized using descriptive statistics. Proportions were compared using Fischer's exact test. Within group and between-groups comparisons were done using *t*-test. Global assessment for efficacy and tolerability was done by comparing the proportion of patients showing excellent and good response against proportion of patients showing fair and poor response. For all statistical tests, a *P* value of less than or equal to 0.05 was considered as significant, after correction for any multiple comparisons.

## 3. Results

**Figure 1:**
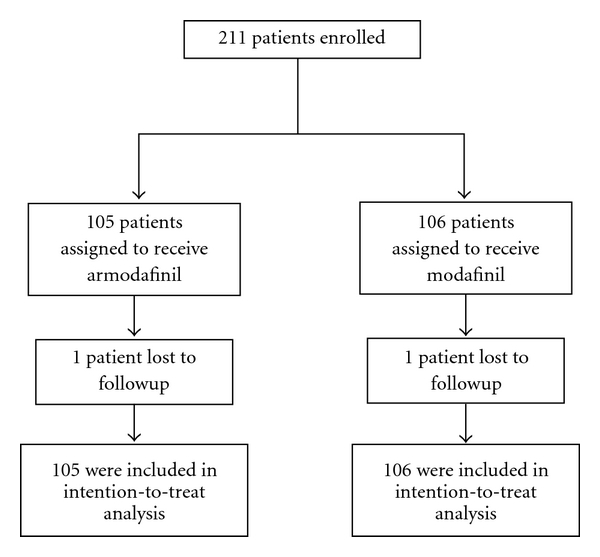
Study flow chart.

**Figure 2:**
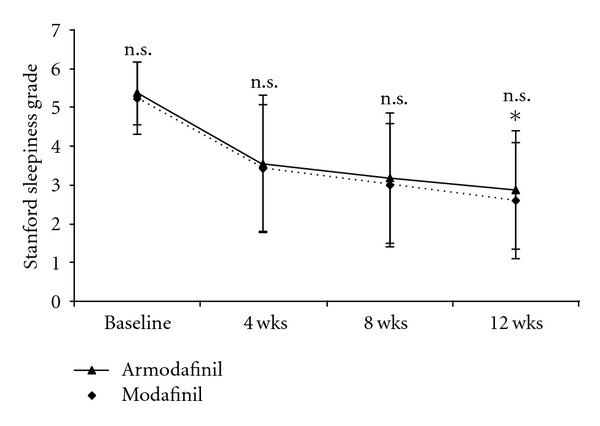
Improvement in Stanford sleepiness scale. *P < .0001 at 12 wks versus baseline for both modafinil and armodafinil using paired *t* test; n.s.: not significant intergroup differences at baseline, 4, 8, and 12 weeks using unpaired *t*-test.

**Figure 3:**
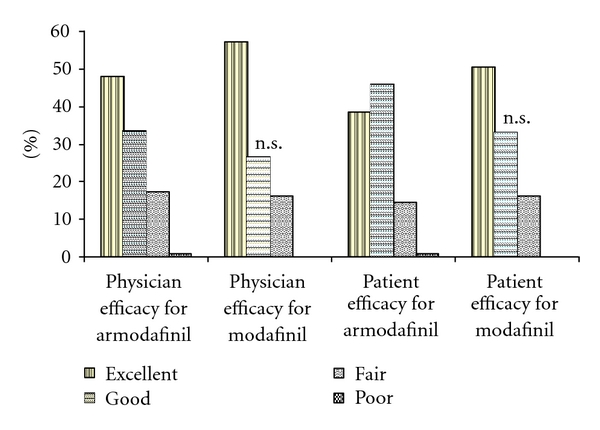
Physicians' and patients' assessment of efficacy. Patients with good and excellent response in armodafinil group were compared against those in modafinil group using Fischer exact test; n.s.—not significant; (excellent—reduction of >75% of symptoms, good: reduction of 51–75% of symptoms, fair: reduction of 26–50% of symptoms, poor: no improvement or reduction in <25% of symptoms).

Two hundred and eleven patients of SWSD were recruited with 105 subjects in armodafinil group and 106 subjects in the modafinil group (Figure 1). The baseline demographic parameters of both groups were comparable (Table 2). Both modafinil and armodafinil significantly improved sleepiness grades as compared to baseline (*P* < .0001) (Figure 2). Responder rates with armodafinil (72.12%) and modafinil (74.29%) were comparable (*P* = .76). At the end of therapy, compliance in both modafinil group (99.31% ± 3.06%) and armodafinil group (99.13% ± 2.35%) was found to be good and comparable (*P* = .63) indicating adequate patient adherence to therapy. Both physicians' and patients' assessment of efficacy was found to be comparable between armodafinil and modafinil group (Figure 3). The intention-to-treat analysis showed that the adverse event incidences in modafinil (40.57%) and armodafinil (42.87%) groups were similar (*P* = .78). 

**Figure 4:**
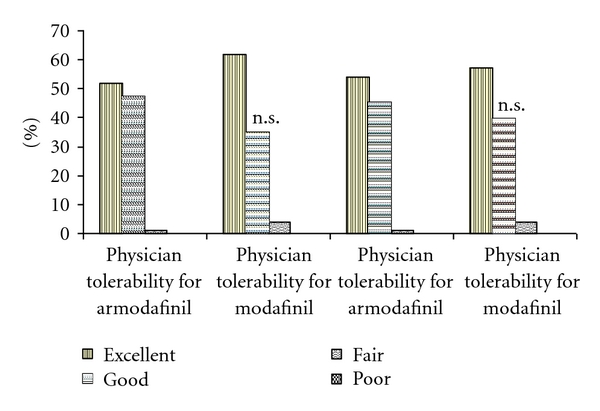
Physicians' and Patients' Assessment of Tolerability. Patients with good and excellent response in armodafinil group were compared against those in modafinil group using Fischer exact test; n.s.: Not significant; (Excellent: No Adverse drug reaction, Good: Mild Adverse drug reaction. No interference with normal lifestyle, Fair: Mild Adverse drug reaction which interference with normal lifestyle. Benefit outweighs inconvenience, Poor: Drug withdrawn).

The adverse effect profile of both drugs was found to be similar with headache, nausea, and dry mouth being the common adverse effects (Table 3). There were no serious adverse events reported during the study. No adverse effects on cognitive or psychomotor functions reported during the study. No incidences of accidents or absenteeism from work were noted during the study period as assessed from patient history. Physicians' and patients' assessment of tolerability was found to be comparable between armodafinil and modafinil group (Figure 4). The baseline and after-therapy biochemical values were within normal range and similar between two groups, except that there was slight increase in mean SGPT in both armodafinil and modafinil groups as compared to baseline (*P* = .008 and  .0007) without inter-group significance and mean blood urea value in armodafinil group increased (*P* = .002) compared to baseline. However, the increased values were within normal limits. In both groups, electrocardiograms were within normal at baseline and after completion of therapy in all patients. One patient in each group opted to discontinue therapy due to adverse events. The adverse events that led to discontinuation were palpitation, anxiety, hypertension, depression, nervousness, and depressed mood in a patient receiving armodafinil and vomiting along with dizziness in another patient receiving modafinil.

## 4. Discussion

The present study confirms the efficacy of armodafinil 150 mg in patients of SWSD. The efficacy of armodafinil was found to be comparable to 200 mg of modafinil in maintaining wakefulness. The safety profile of armodafinil was found to be similar to modafinil. Both modafinil and armodafinil caused a slight increase in liver enzymes, and armodafinil caused a slight increase in blood urea nitrogen. This was not of clinical significance as the increased values were within normal laboratory limits [13]. Armodafinil 150 mg was comparable to modafinil 200 mg, which indicates that armodafinil is 1.33 time more potent than racemic modafinil. The use of R-enantiomer of modafinil avoids unnecessary use of S-isomer and exerts less metabolic load on the body. 

In previous studies, 200 mg of armodafinil was shown to provide more sustained plasma concentrations late in the day as compared to 200 mg of modafinil and monophasic plasma elimination kinetics as compared biphasic for modafinil [8]. This was due initial faster elimination of the S-isomer of modafinil. This pharmacokinetic advantage was claimed to translate into therapeutic benefit. We chose the 150 mg dose of armodafinil, as this was the approved dosage for the present indication. Our study demonstrated no difference in the efficacy of 150 mg of armodafinil over 200 mg of modafinil. The comparative efficacy of 200 mg of armodafinil with modafinil in SWSD has not yet been assessed. 

A limitation of the present study is that the assessment of sleep latency and polysomnography throughout the nightshift could not be done due to unavailability of patients and investigators. This prevented assessment of the clinical correlates of pharmacokinetic advantages of armodafinil [7].

## 5. Conclusion

Armodafinil was found to be safe and effective in the treatment of SWSD. The study did not demonstrate any difference in efficacy and safety between armodafinil 150 mg and modafinil 200 mg, and both drugs were comparable.

##  Disclosure

The study including investigational products was sponsored by Emcure Pharmaceuticals Ltd., Pune, India. The authors received a research grant from Emcure Pharmaceuticals Ltd. for this study.

## Figures and Tables

**Table 1:** 

Diagnostic criteria for SWSD (adopted from ICSD criteria)^3^	
(A) The patient has a primary complaint of insomnia or excessive sleepiness.	
(B) The primary complaint is temporally associated with a work period (usually night work) that occurs during the habitual sleep phase.	
(C) No medical or mental disorder accounts for the symptoms.	
(D) The symptoms do not meet criteria for any other sleep disorder producing insomnia or excessive sleepiness (e.g., time-zone change (jet lag) syndrome).	
Minimal criteria: A plus B.	

**Table 2:** Baseline and demographic parameters.

Parameter	Armodafinil group	Modafinil group	*P*
Number of patients	105	106	NA
Age in years (Mean ± SD)	32.15 ± 10.25	31.90 ± 9.35	.85
Weight (Kg) (Mean ± SD)	60.88 ± 11.43	61.09 ± 11.33	.52
Male: female	81 : 24	90 : 16	.16*
Total number of months working in night shift (mean ± SD)	44.44 ± 119.53	36.41 ± 42.10	.52
Systolic BP mm Hg (mean ± SD)	122.83 ± 9.67	123.39 ± 11.38	.70
Diastolic BP mm Hg (mean ± SD)	78.83 ± 6.75	78.16 ± 7.16	.49

All statistical tests were unpaired *t*-test except for the * = Fisher's exact test; *P* < .05 = statistically significant. NA: Not applicable.

**Table 3:** Intention-to-treat analysis of adverse events in 211 patients.

	Armodafinil *n* (%)	Modafinil *n* (%)	*P*
Cardiovascular			
Palpitation	6 (5.71)	9 (8.49)	.59
Hypertension	4 (3.81)	8 (7.55)	.37
Angina	0 (0)	0 (0)	NA

Dermatologic			
Skin rash	1 ( 0.95)	0 (0)	.48
Contact dermatitis	0 (0)	0 (0)	NA

Gastrointestinal			
Nausea	13 (12.38)	11 (10.38)	.67
Vomiting	1 (0.95)	2 (1.89)	1.00
Dry mouth	15 (14.29)	19 (17.92)	.58
Dyspepsia	6 (5.71)	9 (8.49)	.59
Constipation	11 (10.48)	5 (4.72)	.13
Abdominal pain	4 (3.81)	5 (4.72)	1.00
Diarrhea	0 (0)	4 (3.77)	.12

Psychiatric			
Insomnia	5 (4.76)	11 (10.38)	.20
Anxiety	7 (6.67)	9 (8.49)	.80
Depression	2 (1.90)	0 (0)	.25
Agitation	3 (2.86)	6 (5.66)	.50
Nervousness	10 (9.52)	4 (3.77)	.10
Depressed mood	4 (3.81)	0 (0)	.06

Neurological			
Dizziness	2 (1.90)	8 (7.55)	.10
Disturbance in attention	3 (2.86)	2 (1.89)	.68
Tremor	3 (2.86)	7 (6.60)	.33
Headache	14 (13.33)	15 (14.15)	1.00
Migraine	1 (0.95)	0 (0)	.48
Paraesthesia	0 (0)	0 (0)	NA

General			
Fatigue	4 (3.81)	4 (3.77)	1.00
Thirst	12 (11.43)	6 (5.66)	.15
Influenza like illness	0 (0)	0 (0)	NA
Fever	1 (0.95)	0 (0)	.48
Total no. of patients with adverse events	45 ( 42.85)	43 (40.57)	.78

Fisher's exact test; *P* < .05 = statistically significant.
